# Impact of Statin Therapy on Clinical Outcomes of Patients Hospitalised with Skin and Soft Tissue Infections

**DOI:** 10.3390/jcm15103755

**Published:** 2026-05-13

**Authors:** Ying Chuin Wee, Udul Hewage, Chris Horwood, Yogesh Sharma

**Affiliations:** 1 Department of Acute and General Medicine, Flinders Medical Centre, Bedford Park, SA 5042, Australia; udul.hewage@sa.gov.au (U.H.); yogesh.sharma@sa.gov.au (Y.S.); 2 Clinical Improvement Unit, Flinders Medical Centre, Bedford Park, SA 5042, Australia; chris.horwood@sa.gov.au; 3 College of Medicine & Public Health, Flinders University, Bedford Park, SA 5042, Australia

**Keywords:** length of stay, mortality, septic shock, soft tissue infections, statin

## Abstract

**Background**: Statins have anti-inflammatory properties beyond their lipid-lowering effects, but their impact on skin and soft tissue infections (SSTIs) remains unclear. This study evaluated whether statins improve clinical outcomes in patients hospitalised with SSTIs. **Methods**: Adults aged ≥18 years hospitalised with SSTIs at a tertiary hospital in Australia between 1 June 2021 and 31 December 2021 were identified using the International Classification of Diseases 10th Revision Australian Modification (ICD-10-AM) codes. Patients were categorised into two groups based on statin use at admission. Multivariable regression models assessed differences in clinical outcomes including length of hospital stay (LOS), septic shock, Medical Emergency Response Team (MET) calls, intensive care unit (ICU) admission, mortality and 30-day readmissions, adjusting for age, sex, Charlson Comorbidity Index and C-reactive protein levels. **Results**: Of 387 admissions, complete data were available for 381 patients. The mean (standard deviation [SD]) age was 58.5 years (21.9 years), and 55.9% were male. Statins were used by 110 patients (28.9%) at admission. Statin users were older, had more comorbidities, and were more likely to have positive culture results than statin non-users (*p* < 0.05). Median (interquartile range [IQR]) LOS was significantly longer for statin users compared to non-users (4 [2, 7] versus 3 [2, 5] days, *p* < 0.05). However, after adjusted analysis, LOS was not significantly different between the two groups (adjusted incidence risk ratio [aIRR] 1.08; 95% confidence interval [CI] 0.97–1.20, *p* = 0.134). Other clinical outcomes were also similar between the two groups. **Conclusions**: This study found that statin use at admission was not associated with statistically significant differences in clinical outcomes among patients hospitalised with SSTIs.

## 1. Introduction

Skin and soft tissue infections (SSTIs) are common conditions that frequently lead to hospitalisations, imposing a considerable burden on healthcare systems due to high morbidity and associated costs, with an estimated total direct healthcare expenditure of $15 billion in the United States as of 2012 [1,2,3]. In Australia, while comprehensive national data are limited, a study within the Monash Health network in Victoria estimated an average cost of AUD 16,528 per hospital admission for SSTIs among people who inject drugs, highlighting the significant economic impact on the healthcare system [4]. The management of SSTIs typically involves antimicrobial therapy and supportive care, though patient outcomes can vary depending on individual characteristics and existing comorbidities [5].

Statins, primarily known for their lipid-lowering effects, have also been recognised for their pleiotropic properties, including anti-inflammatory and immunomodulatory effects [6,7]. Statins mediate their anti-inflammatory effects primarily by inhibiting the mevalonate pathway, which decreases the synthesis of isoprenoids [6]. This leads to reduced prenylation of proteins involved in inflammatory signalling, like NF-κB (Nuclear Factor kappa-light-chain-enhancer of activated B cells), thus lowering the production of pro-inflammatory cytokines [6]. Furthermore, statins suppress the expression and activity of Toll-like receptors (TLRs), particularly TLR2 and TLR4, resulting in a shift in immune responses towards an anti-inflammatory state [8]. Given these properties, there is growing interest in exploring whether statins could positively influence clinical outcomes in patients with infections, including SSTIs.

Several studies have suggested that statin use may reduce the risk of sepsis, septic shock, length of hospital stay (LOS), intensive care unit (ICU) admission and mortality in patients with bacterial infections [9,10,11,12,13,14]. For instance, Almog et al. demonstrated a significant reduction in the risk of severe sepsis and ICU admissions among statin users hospitalised with acute bacterial infections. Specifically, severe sepsis occurred in only 2.4% of the statin group compared to 19% in the non-statin group, yielding an 87% relative risk reduction (*p* < 0.001), while ICU admissions were similarly lower among statin users (3.7% vs. 12.2%, *p* = 0.025) [10]. Nassaji et al. supported these findings, observing both reduced mortality and shorter hospital stays in patients with acute bacterial infections on statin therapy [9]. Ghayda et al., in an umbrella review of meta-analyses, found that statin use was associated with reduced mortality in infections such as bacteraemia, sepsis, and pneumonia, though most findings were based on weak evidence [11]. In addition, Liappis et al. reported a 22-percentage-point absolute mortality reduction in bacteraemic patients on statins and Policardo et al. noted a lower hospitalisation risk for bacterial infections in patients on statins irrespective of diabetes status [12,13]. However, evidence on the impact of statins specifically on SSTI outcomes remains limited and inconsistent. While some studies indicate a potential protective effect, others report no significant benefits [9,10,11,12,13,14,15,16]. In the Australian healthcare setting, studies on statin effectiveness in SSTIs are especially sparse, highlighting the need for further investigation.

To address this gap, we conducted a retrospective observational study to examine the association between statin use at hospital admission and clinical outcomes in patients hospitalised with SSTIs. Given statins’ potential immunomodulatory and anti-inflammatory effects, we hypothesised that patients on statins would experience improved outcomes, including a shorter LOS and reduced rates of septic shock, Medical Emergency Response Team (MET) calls, ICU admissions, mortality and readmission within 30 days post-discharge.

## 2. Materials and Methods

This retrospective study included data of all adults aged 18 years and older who were hospitalised with SSTIs at Flinders Medical Centre, Adelaide, between 1 June 2021 and 31 December 2021. Patients were identified using the ICD-10-AM codes specific to SSTIs including L00 (Staphylococcal scalded skin syndrome), L01 (Impetigo), L02 (Cutaneous abscess, furuncle, and carbuncle), L03 (Cellulitis and acute lymphangitis), L04 (Acute lymphadenitis), L05 (Pilonidal cyst and sinus) and L08 (Other local infections of skin and subcutaneous tissue) [17].

Data were collected from the Electronic Medical Records (EMRs), capturing demographic information such as age, gender, ethnicity, residential status, and LOS. Medication details were recorded focusing on statin use at admission, specific statins used and type of antibiotics administered. We gathered details on vital signs (respiratory rate, oxygen saturation, blood pressure, heart rate and temperature) first recorded at admission and blood test results, including white cell count, neutrophils, CRP, creatinine levels, and blood culture results. Relevant comorbidities were documented, and the Charlson Comorbidity Index (CCI) was calculated [18]. We recorded various clinical outcomes including LOS adjusted for in-hospital mortality, incidence of septic shock, occurrence of MET calls, ICU admission during the hospital stay, inpatient mortality, and readmissions within 30 days post-discharge.

### 2.1. Statistical Analysis

The normality of variables was assessed by visual inspection of histograms. Patients were categorised into two groups based on statin exposure. Statin exposure was defined based on medication use at the time of hospital admission. Data regarding continuation or discontinuation of statins during hospitalisation were not available. Continuous variables are presented as means with standard deviations (SDs) or medians with interquartile ranges (IQRs) and categorical variables as proportions. Continuous variables were compared using *t*-tests or Wilcoxon rank-sum tests, as appropriate, while categorical variables were compared using chi-squared statistics. Multivariable regression models were employed to evaluate differences in clinical outcomes between the two groups, adjusting for age, sex, CCI, and CRP levels. Incidence risk ratios (IRRs) or odds ratios (ORs) with 95% confidence intervals (CI) were calculated. All tests were two-sided and a *p* value < 0.05 was considered statistically significant. All statistical analyses were conducted using STATA software version 18.0.

### 2.2. Clinical Outcomes

The primary outcome of this study was the difference in LOS between statin users and non-users. Secondary outcomes included the number of MET calls, the risk of ICU admission, the incidence of septic shock, in-hospital mortality and 30-day readmissions.

## 3. Results

**Figure 1:**
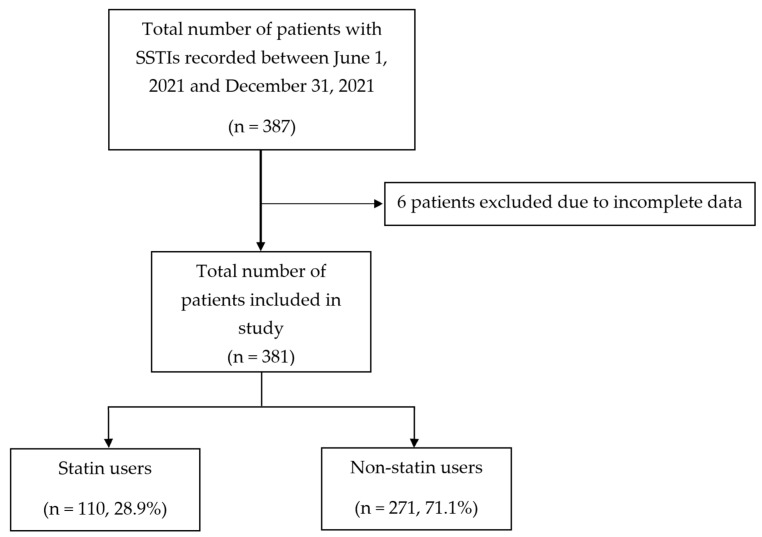
Flow diagram of patients recorded and included in study.

During the study period, a total of 387 admissions with SSTIs were recorded, with complete data available for 381 patients (Figure 1).

Among these patients, 198 patients (52%) presented with cellulitis, while 183 (48%) had other types of SSTIs. The mean age of the cohort was 58.5 years (SD 21.9), and 55.9% were male. Most patients resided at home, while 34 (8.9%) were from a residential facility. Blood cultures were obtained from 120 patients (31.5%), of which 12 (10%) were positive, including 2 false positives due to contamination. The most commonly identified organisms were *Streptococcus* species (60%), followed by *Staphylococcus aureus* (20%) and *Pseudomonas aeruginosa* (20%). One patient had a positive body fluid culture result that grew *Staphylococcus aureus*.

At admission, 110 patients (28.9%) were receiving statins. The most commonly prescribed statin was atorvastatin in 62 patients (56.4%), followed by Rosuvastatin in 32 (29.1%), Simvastatin in 12 (10.9%) and pravastatin in 4 (3.6%) (Table 1). The mean (SD) doses for the prescribed statins are also presented in Table 1.

Baseline characteristics stratified by statin use are summarised in Table 2. Patients receiving statins were significantly older and had a greater burden of comorbidities and cardiovascular risk factors compared with non-users (*p* < 0.05) (Table 2). They also had higher creatinine levels (mean 117.1 µmol/L vs. 82.5 µmol/L, *p* < 0.001) and a higher rate of positive blood or tissue cultures (19.1% vs. 6.3%, *p* = 0.031). Other characteristics, including antibiotic use, were comparable between groups (*p* > 0.05) (Table 2).

### Clinical Outcomes

Clinical outcomes stratified by statin use are presented in Table 3. The median LOS was longer among patients receiving statins compared with non-users (4 days [IQR 2–7] versus 3 days [IQR 2–5], *p* = 0.0004). Other outcomes were similar between the two groups (*p* > 0.05).

In univariable analysis, statin use was associated with an increased risk of a prolonged LOS (IRR 1.58; 95% CI 1.44–1.73; *p* < 0.001). However, after adjusting for age, sex, CCI, and CRP, the association was no longer statistically significant (adjusted incidence risk ratio [aIRR] 1.08; 95% CI 0.97–1.20; *p* = 0.134).

Similarly, no significant associations were observed between statin use and other clinical outcomes, including septic shock (aOR 0.79; 95% CI 0.15–4.12; *p* = 0.786), ICU admission (aOR 2.24; 95% CI 0.18–27.71; *p* = 0.528), MET calls (aOR 0.49; 95% CI 0.11–2.04; *p* = 0.328), total number of MET calls (aIRR 0.64; 95% CI 0.20–2.05; *p* = 0.462), and 30-day readmissions (aOR 1.02; 95% CI 0.48–2.14; *p* = 0.957) (Table 4). However, event rates for these outcomes were low, resulting in wide confidence intervals and limited precision of effect estimates. For example, ICU admission occurred in only four patients overall, and septic shock in eight patients.

## 4. Discussion

This retrospective study found that 28.9% of patients hospitalised with SSTIs were receiving statins at admission. Statin users were older and had a higher burden of comorbidities and cardiovascular risk factors compared with non-users. They were also more likely to have positive culture results, while inflammatory markers were similar between groups. Contrary to our hypothesis, statin use was not associated with improved clinical outcomes. Although unadjusted analysis showed a longer LOS among statin users, this difference was no longer significant after adjustment for confounders. Other clinical outcomes were similarly comparable between groups. However, these findings should be interpreted with caution. The low number of events for several outcomes—particularly ICU admission, septic shock, and mortality—substantially limited statistical power and resulted in wide confidence intervals. As such, the absence of statistically significant associations does not exclude the possibility of clinically meaningful effects of statin therapy in this population.

To date, no studies have specifically evaluated the LOS of statin users hospitalised with SSTIs; however, our findings are consistent with studies investigating statin use in other infectious conditions [19,20,21,22,23]. For example, a meta-analysis examining the effects of statin therapy in hospitalised COVID-19 patients found no significant difference in LOS between statin users and non-users (mean difference [MD] 0.21 days; 95% CI −1.74 to 2.16; *p* = 0.83) [19]. Similarly, a randomised controlled trial (RCT) of atorvastatin in patients with severe sepsis reported no significant difference in LOS between statin and placebo groups [20]. The ASEPSIS RCT, which investigated atorvastatin use in ward patients with sepsis, also found no significant reduction in LOS [21]. Moreover, Havers et al. demonstrated no significant association between statin use and LOS in a prospective cohort study investigating community-acquired pneumonia (CAP), even after adjusting for confounders and applying propensity score analyses. The adjusted hazard ratio (HR) for LOS was 0.99 (95% CI 0.88–1.12), highlighting no clear benefit of statin therapy on this outcome [22]. Sharma et al., in a study conducted at the same centre as the current research, also found no differences in LOS between statin users and non-users among hospitalised CAP patients, after adjustments for age, sex, comorbidities, and pneumonia severity scores [23].

Our findings regarding ICU admission, mortality and hospital readmission rates are also consistent with prior studies showing no significant benefit of statins in acute infections [21,24,25,26]. For instance, a study by Gui et al., which investigated older Chinese patients hospitalised for bacterial infections, found no significant difference in ICU admission rates between statin users and non-users (13.3% vs. 16.8%; *p* = 0.469) [26]. Similarly, the ASEPSIS trial, which examined the effect of atorvastatin on reducing sepsis severity, reported no significant impact on ICU admission and 28-day readmission rates [21]. Furthermore, a meta-analysis by Wan et al., which included five RCTs and 27 observational studies, examined the impact of statins on mortality in patients with infection and sepsis. While the observational studies suggested a significant reduction in mortality among statin users (adjusted RR, 0.65; 95% CI, 0.57–0.75), the randomised trials found no significant difference, indicating limited evidence of statin benefit in this context [24].

Interestingly, statin users had a higher rate of positive blood culture results, despite similar inflammatory markers. This observation raises questions about the factors contributing to culture positivity in statin users. One possible explanation is the higher prevalence of comorbidities, such as diabetes, in this group. Diabetes is known to increase susceptibility to infections, potentially leading to increased rates of bloodstream infections among these patients [27,28]. For example, Thomsen et al. demonstrated a 2.1-fold increased risk of streptococcal bacteraemia among individuals with diabetes compared to controls in a 15-year population-based case–control study in Denmark [27]. Similarly, a retrospective study by Stoeckle et al. revealed that diabetic patients had a 4.4-fold increased incidence of bloodstream infections compared to non-diabetics [28]. The presence of other chronic health conditions such as CKD and CCF among statin users may also predispose them to more severe or systemic infections [29,30]. Another possibility is that statins themselves may have adverse effects on infection risk. For example, Ko et al. conducted a sequence symmetry analysis and found that statin users, regardless of diabetes status, had a significantly increased risk of SSTIs (adjusted sequence ratio > 1; confidence interval > 1), indicating that statins may independently contribute to the heightened risk of these infections [15]. However, this finding in our cohort is based on a very small number of events (n = 13), and this finding should be interpreted with caution as it may reflect a chance association rather than a true underlying effect.

Some studies have linked statin therapy to a reduction in inflammatory markers, particularly CRP [31,32,33]. Notably, the Pravastatin Inflammation/CRP Evaluation (PRINCE) study demonstrated that pravastatin significantly reduced CRP levels in a manner largely independent of low-density lipoprotein cholesterol (LDL-C) reduction, with a median CRP reduction of 16.9% observed at 24 weeks in patients receiving pravastatin compared to placebo [31]. A comprehensive meta-analysis also showed that statins, on average, led to a significant reduction in CRP levels, with a reduction of −0.65 mg/L (95% CI −0.87 to −0.43) compared to controls [32]. Furthermore, a study by Chalmers et al. (2008) reported that statin use in patients with community-acquired pneumonia was associated with significantly lower CRP levels upon admission (119 mg/L vs. 182 mg/L, *p* < 0.0001), suggesting an anti-inflammatory effect of statins even in infection-related inflammation [33]. However, our study did not observe this effect. This discrepancy raises questions about the consistency and magnitude of the immunomodulatory effects of statins, particularly in patients with SSTIs, where the inflammatory burden may differ from that in cardiovascular conditions or other infections. It is possible that the anti-inflammatory effects of statins are insufficient to meaningfully influence CRP levels in the context of SSTIs, especially in patients with significant comorbidities.

The types and doses of statins used in our cohort were comparable to previous studies, with atorvastatin being the most commonly prescribed agent [9,13]. While higher statin doses have been associated with greater reductions in inflammatory markers, their impact on infection-related outcomes remains unclear [34].

### 4.1. Strength

This study contributes to the existing evidence on statin use in infectious diseases, specifically focusing on hospitalised patients with SSTIs, a topic that remains relatively underexplored especially in the Australian healthcare setting. The use of a well-defined cohort and adjustment for key confounders, including age, comorbidity burden and inflammatory markers, strengthens the validity of our findings. Additionally, prespecification of secondary outcomes reduces the risk of outcome reporting bias.

### 4.2. Limitations

As a retrospective study, the reliance on electronic medical records (EMRs) can introduce inherent selection bias and misclassification. While EMRs provide large-scale data access, they are subject to documentation errors and variability in clinical recording practices. Additionally, the observational nature of the study precludes the establishment of causality.

Despite adjustment for key covariates, residual confounding remains a concern. Statin users in our cohort were substantially older and had a higher comorbidity burden, which may reflect differences in underlying health status not fully captured by the Charlson Comorbidity Index. In particular, unmeasured factors such as frailty, severity of infection at presentation, delays in seeking care, duration of statin use prior to admission and in-hospital continuation or discontinuation of therapy may have influenced outcomes. Although multivariable regression was used to adjust for measured confounders, residual confounding cannot be excluded.

The study was also underpowered for several secondary outcomes due to low event rates, including ICU admission, septic shock, and mortality. Consequently, the absence of statistically significant associations should not be interpreted as definitive evidence of no effect, particularly given the wide confidence intervals around some estimates.

We also did not stratify statin therapy according to treatment intensity (e.g., high- versus moderate-/low-intensity). Differences in statin dose and potency may influence their biological effects, and this could not be assessed in the present study due to the limited sample size and event rates.

Furthermore, as a single-centre study conducted in a tertiary care hospital, the results may not be generalisable to other hospitals or healthcare settings. The study was conducted over a relatively short period (June–December 2021), which may introduce potential seasonal bias, as the epidemiology and severity of SSTIs can vary across different times of the year. Consequently, the findings may not fully capture seasonal variation in SSTI presentations and outcomes.

Future prospective multicentre studies with larger sample sizes and evaluation of different statin types, dosages and treatment durations are needed to better clarify the potential role of statins in SSTIs and other infectious diseases.

## 5. Conclusions

Our study did not find evidence to support a significant benefit of statin use in improving clinical outcomes in adults hospitalised with SSTIs. While statin therapy was associated with a longer unadjusted LOS, this effect was not significant after adjusting for confounding variables, suggesting that baseline patient characteristics drive observed differences. Larger prospective, multicentre studies are needed to further evaluate the role of statins in SSTIs and other infectious diseases.

## Figures and Tables

**Table 1:** Types and dosages of statins used.

Type	N (%)	Mean (SD) Dose in mg	Dose Range in mg
Atorvastatin	62 (56.4)	39.2 (24.1)	10–80
Rosuvastatin	32 (29.1)	14.5 (9.2)	5–40
Simvastatin	12 (10.9)	41.7 (24.8)	20–80
Pravastatin	4 (3.6)	17.5 (5.0)	10–20

Abbreviations: SD—standard deviation.

**Table 2:** Baseline characteristics of patients stratified by statin use.

Variable	Not on Statins	On Statins	*p* Value
Total number n (%)	271 (71.1)	110 (28.9)	
Age mean (SD)	52.2 (22.0)	73.3 (13.1)	<0.001
Age group n (%)			<0.001
<40	92 (33.9)	2 (1.8)	
40–59	82 (30.3)	15 (13.6)	
60–79	57 (21.0)	56 (50.9)	
>80	40 (14.8)	37 (33.6)	
Male sex n (%)	141 (52.0)	72 (65.5)	0.051
Residential status home n (%)	248 (91.5)	99 (90.0)	0.639
Ethnicity n (%)			0.390
Caucasians	259 (95.6)	108 (98.2)	
Indigenous	9 (3.3)	2 (1.8)	
Others	3 (1.1)	0 (0)	
Diabetes n (%)	39 (14.4)	54 (49.1)	<0.001
Obesity n (%)	71 (51.5)	38 (47.5)	0.574
Hypercholesterolaemia n (%)	20 (7.4)	102 (92.7)	<0.001
Hypertension n (%)	74 (27.3)	88 (80.0)	<0.001
PVD n (%)	14 (5.2)	24 (21.8)	<0.001
CHF n (%)	29 (10.7)	34 (30.9)	<0.001
Myocardial infarction n (%)	14 (5.2)	33 (30.0)	<0.001
Stroke/TIA n (%)	5 (1.9)	18 (16.4)	<0.001
Dementia n (%)	14 (5.2)	10 (9.1)	0.153
CKD n (%)	8 (2.9)	11 (10.0)	0.004
Cancer n (%)	34 (12.6)	23 (20.9)	0.038
Immunosuppression n (%)	103 (50.2)	39 (51.3)	0.873
CCI mean (SD)	2.4 (2.9)	5.8 (2.9)	<0.001
Smokers n (%)	103 (50.2)	39 (51.3)	0.873
WBC × 10^9^/L mean (SD)	11.9 (12.9)	10.8 (4.7)	0.418
Neutrophil × 10^9^/L mean (SD)	8.0 (4.6)	8.4 (4.6)	0.527
CRP mg/L median (IQR)	25.2 (10–73.8)	31.9 (7.3–91.7)	>0.05
Creatinine µmol/L mean (SD)	82.5 (60.3)	117.1 (96.5)	<0.001
Positive culture blood/tissue n (%)	5 (6.3)	8 (19.1)	0.031
Flucloxacillin n (%)	99 (36.5)	50 (45.4)	0.246
Cephazolin n (%)	101 (37.3)	42 (38.2)	0.722
Vancomycin n (%)	29 (10.7)	8 (7.3)	0.545
Other antibiotics n (%)	89 (32.8)	39 (35.4)	0.685

Abbreviations: PVD—peripheral vascular disease; CHF—congestive heart failure; TIA—transient ischaemic attack; CKD—chronic kidney disease; CCI—Charlson Comorbidity Index; WBC—white blood cell; CRP—C-reactive protein; SD—standard deviation; IQR—interquartile range.

**Table 3:** Clinical outcomes based on statin use status.

Outcome	Not on Statins	On Statins	*p* Value
LOS median (IQR) *	3 (2–5)	4 (2–7)	0.0004
MET call n (%)	8 (2.9)	3 (2.7)	0.904
Total no of MET calls mean (SD)	0.01 (0.57)	0.05 (0.31)	0.720
ICU admission	2 (0.7)	2 (1.8)	0.349
Septic shock	5 (1.9)	3 (2.7)	0.586
In-hospital mortality	2 (0.7)	0	0.505
30-day readmissions	37 (13.7)	18 (16.4)	0.495

Abbreviations: LOS—length of stay; no—number; MET—medical emergency team; ICU—intensive care unit; IQR—interquartile range; SD—standard deviation. * LOS adjusted for in-hospital mortality.

**Table 4:** Univariable and multivariable regression analysis comparing outcomes based on exposure to statins.

Outcome	Unadjusted OR	95% CI	*p* Value	Adjusted OR *	95% CI	*p* Value
LOS	1.58 †	1.44–1.73	<0.001	1.08 †	0.97–1.20	0.134
MET calls	0.92	0.23–3.54	0.906	0.49	0.11–2.04	0.328
Total no of MET calls	0.68 †	0.25–1.84	0.453	0.64 †	0.20–2.05	0.462
ICU admission	2.49	0.34–17.90	0.365	2.24	0.18–27.71	0.528
Septic shock	1.49	0.35–6.35	0.589	0.79	0.15–4.12	0.786
30-day readmissions	1.23	0.67–2.28	0.496	1.02	0.48–2.14	0.957

* Regression models adjusted for age, sex, Charlson Comorbidity Index and CRP (C-reactive protein). † Incidence risk ratio. Abbreviations: LOS—length of stay; no—number; MET—medical emergency team; ICU—intensive care unit; OR—odds ratio; CI—confidence interval.

## Data Availability

The original contributions presented in this study are included in the article. Further inquiries can be directed to the corresponding author.
